# Microfluidic technology and simulation models in studying pharmacokinetics during pregnancy

**DOI:** 10.3389/fphar.2023.1241815

**Published:** 2023-08-17

**Authors:** Ananth K. Kammala, Lauren S. Richardson, Enkhtuya Radnaa, Arum Han, Ramkumar Menon

**Affiliations:** ^1^ Division of Basic Science and Translational Research, Department of Obstetrics and Gynecology, The University of Texas Medical Branch at Galveston, Galveston, TX, United States; ^2^ Department of Electrical and Computer Engineering, Texas A&M University, College Station, TX, United States; ^3^ Department of Biomedical Engineering, Texas A&M University, College Station, TX, United States

**Keywords:** organ on chip, simulation model, drug transportation and pregnancy pharmacokinetics, PBPK, pregnancy modeling pregnancy pharmacokinetics by microfluidics, simulation, artificial intelligence, mouse models

## Abstract

**Introduction:** Preterm birth rates and maternal and neonatal mortality remain concerning global health issues, necessitating improved strategies for testing therapeutic compounds during pregnancy. Current 2D or 3D cell models and animal models often fail to provide data that can effectively translate into clinical trials, leading to pregnant women being excluded from drug development considerations and clinical studies. To address this limitation, we explored the utility of in silico simulation modeling and microfluidic-based organ-on-a-chip platforms to assess potential interventional agents.

**Methods:** We developed a multi-organ feto-maternal interface on-chip (FMi-PLA-OOC) utilizing microfluidic channels to maintain intercellular interactions among seven different cell types (fetal membrane-decidua-placenta). This platform enabled the investigation of drug pharmacokinetics in vitro. Pravastatin, a model drug known for its efficacy in reducing oxidative stress and inflammation during pregnancy and currently in clinical trials, was used to test its transfer rate across both feto-maternal interfaces. The data obtained from FMi-PLA-OOC were compared with existing data from in vivo animal models and ex vivo placenta perfusion models. Additionally, we employed mechanistically based simulation software (Gastroplus®) to predict pravastatin pharmacokinetics in pregnant subjects based on validated nonpregnant drug data.

**Results:** Pravastatin transfer across the FMi-PLA-OOC and predicted pharmacokinetics in the in silico models were found to be similar, approximately 18%. In contrast, animal models showed supraphysiologic drug accumulation in the amniotic fluid, reaching approximately 33%.

**Discussion:** The results from this study suggest that the FMi-PLA-OOC and in silico models can serve as alternative methods for studying drug pharmacokinetics during pregnancy, providing valuable insights into drug transport and metabolism across the placenta and fetal membranes. These advanced platforms offer promising opportunities for safe, reliable, and faster testing of therapeutic compounds, potentially reducing the number of pregnant women referred to as “therapeutic orphans” due to the lack of consideration in drug development and clinical trials. By bridging the gap between preclinical studies and clinical trials, these approaches hold great promise in improving maternal and neonatal health outcomes.

## 1 Introduction

Pregnancy is a unique physiological condition that can significantly affect the pharmacokinetics of therapeutic drugs (Costantine, 2014; Feghali et al., 2015). Various physiological changes occur during pregnancy, such as increased maternal fat, total body water, blood volume, cardiac output, blood flow to the kidneys, and decreased plasma protein concentrations. These changes make it challenging to predict the pharmacokinetics, mechanism of action and efficacy of drugs during different trimesters of pregnancy (Feghali et al., 2015). Therapeutic interventions during pregnancy are commonly used to treat maternal pathologies such as preeclampsia and intrauterine growth retardation or to delay preterm labor (Cleary et al., 2014).

As one of the feto-maternal interfaces (FMi) during pregnancy, the placenta is the primary organ involved in drug transportation and metabolism (Garnica and Chan, 1996; Gulati and Gerk, 2009; Al-Enazy et al., 2017). However, the fetal membranes surrounding the intrauterine cavity also express transporter proteins (e.g., P-gp, BCRP-1, and OATPs) and metabolic enzymes (CYP P450 enzymes) (Ganguly et al., 2021; Kammala et al., 2021; Kammala AK. et al., 2022; Kammala A. et al., 2022). These membranes are involved in drug propagation and metabolism. Both the placenta and fetal membranes act as protective barriers while facilitating communication and nutrient exchange between the mother and fetus (Behnia et al., 2015; Behnia et al., 2016; Ayad et al., 2018; Menon et al., 2019; Monsivais et al., 2020). Understanding drug transportation and metabolism across these FMis is crucial since pregnant women often take different medications at different gestational stages. However, several limitations exist when studying drug pharmacokinetics in pregnant subjects, including the exclusion of pregnant women from clinical trials, the lack of animal models that mimic human pregnancy, and the high cost of non-human primate models.

Advancements in microfluidic technology have led to the development of micro-physiological systems, such as tissue chips or organ-on-chip models, that mimic various reproductive organs, including the placenta (Blundell et al., 2016; Blundell et al., 2018; Mosavati et al., 2020; Richardson et al., 2022), fetal membranes (Richardson et al., 2019; Richardson et al., 2020a; Richardson L. et al., 2020), cervix (Tantengco et al., 2021), and vagina (Mahajan et al., 2022). Several "placental organ-on-chip” devices have been developed to study drug transportation during pregnancy. However, these devices focus solely on the placental tissue and neglect the fetal membranes. Recent studies have compared the pharmacokinetics and efficacy of drugs using separate placental and fetal membrane interfaces and reported similar transfer rates, metabolism, and therapeutic effects (Basraon et al., 2012; Dursun et al., 2014; Basraon et al., 2015; Ahmed et al., 2020). However, whether these results hold true for the entire placenta-fetal membrane system *in utero* remains unknown.

In addition to microfluidic technology, *in silico* simulation software such as Gastroplus^®^ has proven useful in predicting the pharmacokinetic parameters of chemical compounds using mathematical algorithms (Zhang and Unadkat, 2017; Zurlinden and Reisfeld, 2017). Recent developments in predictive models of drug pharmacokinetics have expanded to include the pregnant population. Physiologically based pharmacokinetic (PBPK) models integrate both the physicochemical properties of drugs and the physiological parameters of subjects, considering the anatomical, physiological, and physical events involved in drug absorption, distribution, metabolism, and excretion (ADME) (De Buck and Mackie, 2007; Espié et al., 2009). Due to the limitations of clinical and basic science approaches, simulating the pharmacokinetics of drugs during pregnancy using *in silico* simulation software provides valuable insights to clinicians regarding the fate of drugs in the pregnant population (Weijs et al., 2012; Zurlinden and Reisfeld, 2017).

In this study, we focused on pravastatin sodium as a prototype drug to evaluate drug propagation and simulate its pregnancy pharmacokinetics using a novel fetal membrane-placenta feto-maternal interface on-chip (FMi-PLA-OOC) and Gastroplus^®^ software. Pravastatin is an HMG-CoA reductase inhibitor commonly used to lower lipid levels and reduce the risk of cardiovascular events (Basraon et al., 2012; Costantine et al., 2013; Basraon et al., 2015). It has shown potential benefits in treating preeclampsia during pregnancy (Kumasawa et al., 2011; Ahmed et al., 2020; Kumasawa et al., 2020; Jurisic et al., 2021). Pravastatin exhibits unique pharmacokinetic parameters, including low oral bioavailability and active transport by transporter proteins (Afrouzian et al., 2018). These parameters limit its passage through the placenta (Nanovskaya et al., 2005; Nanovskaya et al., 2013; Zarek et al., 2013). However, the metabolic profile of pravastatin at both FMis is still under investigation. By utilizing microfluidic systems and simulation models, we aimed to determine pravastatin propagation and metabolism across the FMi and simulate its pharmacokinetics during pregnancy. The developed models were validated using available clinical data and compared with *in vivo* pharmacokinetics in pregnant mouse models.

The United States Congress has recently passed the FDA Modernization Act 2.0 (42). This bill allows an applicant for market approval for a new drug to use methods other than animal testing to establish the drug’s safety and effectiveness. Under this bill, these alternative methods may include cell-based assays, organ chips and microphysiological systems, computer modeling, and other human biology-based test methods. These testing are expected to expedite preclinical drug testing and get drugs to clinical trials and subsequently to the market without much delays. Our primary goal is to establish that microfluidic technology combined with *in silico* simulation models can serve as an alternative method for determining drug pharmacokinetics during pregnancy.

## 2 Materials and methods

### 2.1 Institutional review board approval

Placental specimens used for this study were collected from John Sealy Hospital, University of Texas Medical Branch (UTMB) at Galveston, Texas, according to the inclusion and exclusion criteria described below. The placentas were deidentified and considered discarded human specimens; therefore, subject recruitment and consent were not required. The Institutional Review Board (IRB) at UTMB approved the study protocol (UTMB 11–251), and placentas were collected, according to the regulations of the IRB, as an exempt protocol that allowed the use of discarded placentas for fetal membrane and placental research.

### 2.2 Cell preparation and culture

Primary human maternal and fetal cells were collected from the term, not-in-labor, cesarean deliveries and immortalized based on our previous protocols (Radnaa et al., 2021a) to reduce patient-to-patient variability when studying the responses of each cell type within the FMi-PLA-OOC. Human maternal decidual cells (hFM_DEC) were cultured in DMEM/F12 (Mediatech Inc., Manassas, VA, United States) supplemented with 10% FBS, 10% penicillin/streptomycin (Mediatech), and 10% amphotericin B (Sigma-Aldrich, Inc. St. Louis, MO). Human amnion epithelial cells (hFM_AEC) were cultured in KSFM supplemented with bovine pituitary extract (30 μg/mL), epidermal growth factor (0.1 ng/mL), CaCl_2_ (0.4 mM), and primocin (0.5 mg/mL). Human amnion mesenchymal cells (hFM_AMC) were cultured in DMEM/F12 supplemented with 5% FBS, 10% penicillin/streptomycin, and 10% amphotericin B. Human chorion trophoblast cells (hFM_CTC) were cultured in DMEM/F12 supplemented with 0.20% FBS, 0.01 mM β-mercaptoethanol, 0.5% penicillin/streptomycin, 0.3% BSA, 1× ITS-X, 2 μM CHIR99021, 0.05 μM A83-01, 1.5 μg/mL L-ascorbic acid, 50 ng/mL epithelial growth factor, 0.08 mM VPA, and 1× Revitacell (Rock inhibitor/Y27632). All cells were grown at 37°C and 5% CO_2_ until they reached 80%–90% confluency. Immortalized cells have been validated against primary cells previously. Cells under passage 25 were used for experiments. Placental cytotrophoblasts (BeWo cells) purchased from ATCC (Virginia, United States) were used in this study. BeWo cells were cultured in DMEM/F12 (Mediatech, Manassas, VA, United States) supplemented with 10% FBS, 10% penicillin/streptomycin (Mediatech Inc.), and 10% amphotericin B (Sigma-Aldrich). The cells were grown at 37°C and 5% CO_2_ until 80%–90% confluence was achieved. Furthermore, for syncytiotrophoblasts, BeWo cells were plated and maintained in complete DMEM with 25 μM forskolin for at least 48 h in a 37°C, 5% CO_2_ incubator. As reported in the literature, BeWo cells are used to mimic placental trophoblast cells, although this may not be the ideal cell. BeWo cells were chosen for these experiments as no induced pluripotent stem cells or primary cell lines were available to model second or third-trimester placenta cells. Primary human umbilical vein endothelial cells (HUVECs) were isolated as described previously with modifications (Menon et al., 2020). Briefly, fresh cuts were made on both ends of the umbilical cord and a 21-gauge needle was inserted into the vein and clamped with a hemostat. A 20cc syringe containing Hanks was attached to the needle and the vein was washed twice with the Hanks. Then, the other end of the vein was clamped, and the syringe was replaced with a new 10 mL syringe containing collagenase. Collagenase was slowly injected into the horizontally placed vein and transferred to a sterile wide beaker filled with PBS to incubate for 30 min at 37°C. Then, the cord was placed in a 50 mL tube to collect loosened cells from the vein and was washed once with Hanks. The collected cells were centrifuged at ∼1200rpm for 5 min and seeded into a T25 flask. Cells were cultured in complete Medium 199 containing 20% FBS, 0.1 mg/mL heparin, and 300 μg/mL endothelial cell growth supplement.

### 2.3 Device fabrication, cell loading, and experiments

#### 2.3.1 Device design and fabrication

**FIGURE 1:**
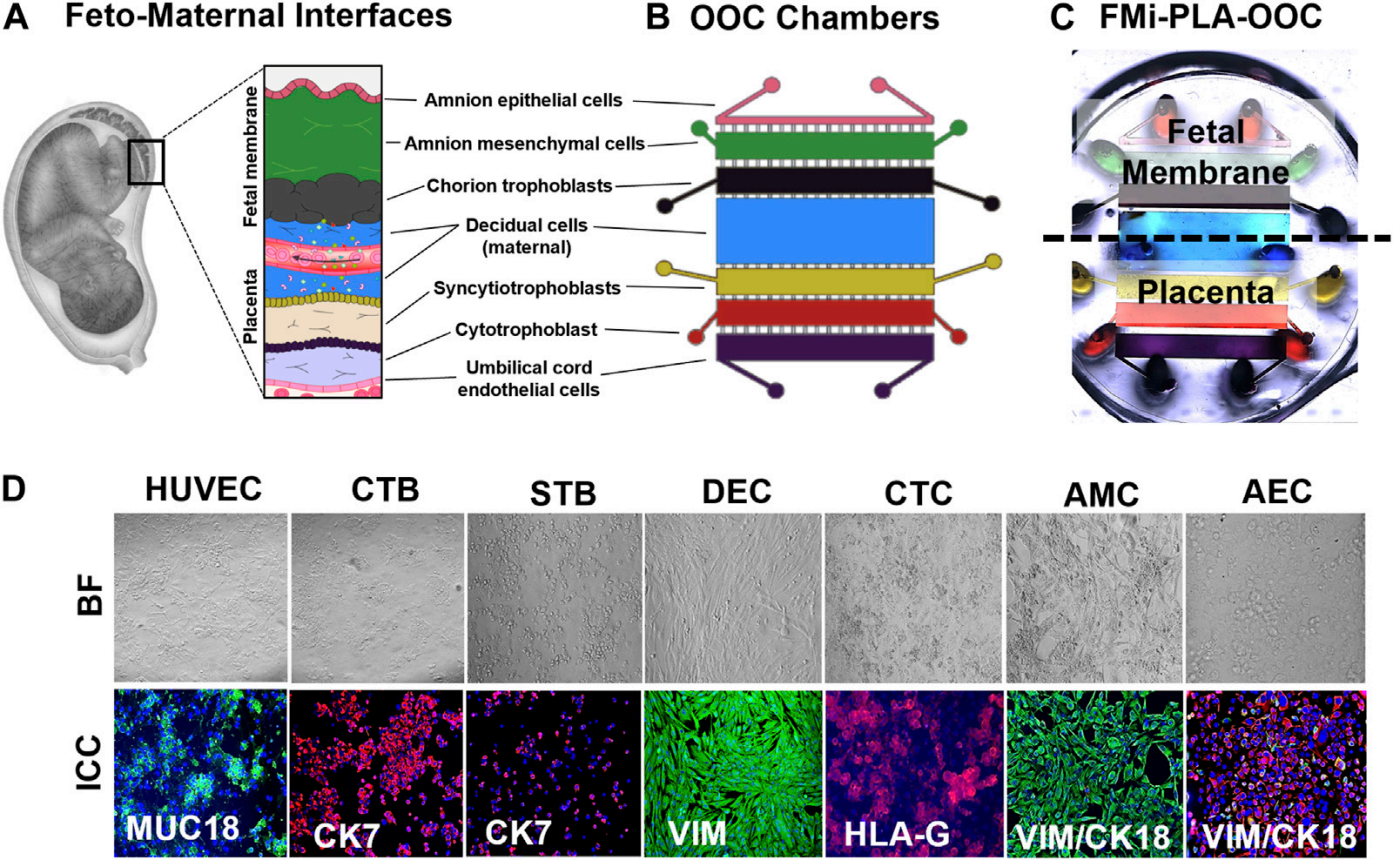
Development of the multi-organ Fetal Membrane-Placenta feto-maternal interface Organ-On-Chip (FMi-PLA-OOC) platform **(A)** Schematic of the intrauterine organs surrounding the fetus, highlighting the two feto-maternal interfaces, the placenta-decidua and fetal membrane-decidua (i.e., fetal-maternal) cellular layers. **(B)** The FMi-PLA-OOC contains seven rectangular cell culture chambers separated by arrays of microchannels. The cells are seeded as follows, from top to bottom: fetal membrane-amnion epithelial cells (AECs) in pink, amnion mesenchymal cells (AMCs) in green, chorion trophoblast cells (CTCs) in black, maternal decidual cells (DECs) in blue, placenta-syncytialized BeWo cells forming the syncytiotrophoblast (STB) layer in yellow, BeWo cells recreating the cytotrophoblast (CTB) layer in red, and human umbilical vein endothelial cells (HUVECs) in purple. Microchannels between the AEC-AMC and AMC-CTC chambers are filled with Type IV collagen to mimic the basement membrane, while the microchannels between the CTB-HUVEC chambers are filled with Type I collagen to mimic the placenta stroma. **(C)** Image of the microfabricated FMi-PLA-OOC filled with color dye for easy visualization of each cell culture chamber. An on-chip media reservoir layer is aligned on top of the cell loading inlets and outlets (ovals) of the main cell culture layer, allowing media perfusion throughout the chamber for 2, 4, or 8 h. Effluents from each culture chamber were collected through the reservoirs. **(D)** A variety of *in utero* characteristics were measured to determine if cells grown within the FMi-PLA-OOC retained their *in vivo* characteristics. These measurements included cell morphology (brightfield microscopy [BF]), cytoskeletal markers (cytokeratin-7 [CK-7]; red) (CK-18; red) (vimentin [Vim]; green); immune regulation receptor (Human leukocyte antigen-G [HLA-G]; red); and endothelial cell marker (MUC18; green) (N = 3).

The microfluidic fetal membrane-placental feto-maternal interface on-chip (FMi-PLA-OOC) is composed of seven poly (dimethylsiloxane) (PDMS) planer rectangular chambers that each form a cell culture chamber modeling both the fetal membrane and placenta FMi during gestation (Figures 1A–C). From left to right, chamber 1 contains fetal HUVEC cells (purple), chamber 2 contains cytotrophoblasts (red), chamber 3 contains syncytiotrophoblasts (yellow), chamber 4 contains maternal decidua (blue), chamber 5 contains fetal chorion trophoblast (black), chamber 6 contains fetal amnion mesenchymal cells (green). Chamber 7 contains fetal amnion epithelial cells (pink) (Figure 1B). Each cell chamber was 250 μm in height, and the width of each chamber was designed to mimic the thickness of each maternal and fetal layer as seen *in utero* (maternal: DEC—3,000 μm [green]; fetal membrane: CTC—2,000 μm, AMC—2,000 μm, and AEC—600 μm) (Richardson et al., 2019; Richardson et al., 2020c; Radnaa et al., 2021b) (placenta: STB—2,000 μm, CTB- 2,000 μm, and HUVEC - 2,000 μm). This design allowed the seven different cell types to be cultured in seven separate microenvironments (e.g., different culture mediums) while maintaining cell-cell and cell-collagen interactions through arrays of microchannels. The chambers were interconnected through an array of 24 microchannels varying in length depending on the application (HUVEC-CTB: 5 μm in height, 30 μm in width, and 600 μm in length filled with Type l collagen to model the placenta stroma; CTB-STB, STB-DEC, DEC-CTC: 5 μm in height, 30 μm in width, and 300 μm in length left empty to form cell-cell connections; and CTC-AMC, AMC-AEC: 5 μm in height, 30 μm in width, and 600 μm in length filled with Type IV collagen to model chorion and amnion basement membrane). The microchannel arrays perform multiple independent functions, including: 1) preventing the flow of cells between compartments during the initial cell loading process, 2) allowing localized treatment of each cell layer with infectious or other stimulants while limiting their diffusion to the adjacent chambers, 3) enabling independent elution of supernatant from each cell chamber, and 4) allowing biochemicals to diffuse between chambers in a time-dependent way and also permit active cell migration that may involve cellular transitions (Richardson et al., 2020d; Tantengco et al., 2021). The device also contains an on-chip reservoir block, having multiple 4 mm diameter and 2 mm deep reservoirs aligned on top of the inlets and outlets of each chamber in the primary cell culture layer (Figure 1C). The designed platform was fabricated in PDMS using a two-step photolithography master mold fabrication process, followed by a soft lithography process of replica molding the final PDMS device from the master mold. First, to create the master mold, two layers of photosensitive epoxy (SU-8; MicroChem, Westborough, MA, United States) with different thicknesses were sequentially patterned on a 3-inch diameter silicon substrate as described with our other chip publications (Richardson et al., 2019; Richardson et al., 2020a; Radnaa et al., 2021b; Tantengco et al., 2021). The master mold was then coated with (tridecafluoro-1,1,2,2-tetrahydro octyl) trichlorosilane (United Chemical Technologies, Bristol, PA, United States) to facilitate PDMS release from the master mold after replication. The PDMS devices were replicated from the master mold by pouring PDMS pre-polymer (1:10 mixture, Sylgard 184; DowDuPont, Midland, MI, United States) on the mold, followed by curing at 85°C for 45–60 min. The reservoirs to hold the culture medium were punched out of a 2 mm thick PDMS block using a 4 mm diameter drill punch. To improve the bonding of the PDMS layer onto the glass substrate and to make the device hydrophilic for easy cell and culture medium loading, the PDMS layers were treated with oxygen plasma (Harrick Plasma, Ithaca, NY, United States) for 90 s, followed by bonding onto a glass substrate. This process was repeated to bond the PDMS reservoir layer on top of the device. The assembled device was then stored dry (for up to 1 month), then sterilization with 70% ethanol for 15 min before use.

#### 2.3.2 Collagen and cell loading

Before using the FMi-PLA-OOC, the devices were washed once with 70% EtOH and twice with PBS, filled with diluted type I (Rat tail Type I collagen; 1:25 in serum-free DMEM/F12 media) and type IV basement membrane collagen Matrigel (Corning Matrigel Basement Membrane Matrix, DEV-free; 1:25 in serum-free DMEM/F12 media), and incubated at 37°C with 5% CO_2_ for 4 h. Specifically, diluted type I basement membrane Matrigel was used to fill the microchannels connecting the HUVECs to CTBs, mimicking the placental basement membrane *in utero*. Diluted type IV basement membrane Matrigel was used to fill the microchannels connecting the AECs to AMCs and AMCs to CTCs, mimicking the amnion and chorion basement membranes *in utero*. Then the CTC and DEC chambers were filled with diluted type IV basement membrane Matrigel, and the STB and HUVEC chambers were filled with diluted type I basement membrane Matrigel. The devices were then incubated for 4 h at 37°C.

#### 2.3.3 Cell seeding and culture in the FMi-PLA-OOC

Before loading the cells, a pipette was used to manually remove the collagen from the chambers of each FMi-PLA-OOC, followed by minimal vacuum pressure to remove any remaining collagen. The devices were filled with complete DMEM media to wash out excess collagen and to keep the devices hydrated. All cell types were passaged and counted to determine the loading number. The media were removed from the devices with a pipette before loading cells. 50,000 DECs, 100,000 CTCs, 50,000 AMCs, 80,000 AECs, 100,000 STBs, 100,000 CTBs, and 80,000 HUVECs were loaded onto each device. Primary collagen and Matrigel were added to the AMC and CTC chambers (CTCs +5% primary collagen +25% Matrigel; AMCs +20% primary collagen +25% Matrigel). Each chamber’s reservoirs were filled with cell-type-specific medium. The devices were placed in a 6-well plate, and PBS was added to the center of the 6-well plate to prevent excessive evaporation from the reservoirs. The devices were incubated at 37°C with 5% CO_2_ overnight. The next day, media from each reservoir were removed and saved for cytotoxicity assays. Media were replaced with a cell-specific medium in each reservoir so that the final gradient within the reservoirs was as follows: HUVEC 25μL/reservoir—CTB 35μL/reservoir—STB 35μL/reservoir—DEC 50μL/reservoir—CTC 35μL/reservoir—AMC 35μL/reservoir—AEC 25μL/reservoir.

#### 2.3.4 Molecular diffusion within the FMi-PLA-OOC

3000k TexasRed Dextran beads were loaded into the DEC chamber to determine diffusion across the fetal membrane and placenta chambers through the interconnected microfluidic channel array to determine media diffusion between chambers over time. The same fluidic gradient was established in the reservoir system, and fluorescent and brightfield microscopy images of the devices were taken every 2-h over a 48-h period. Fluorescence intensity was used to measure the degree of diffusion from chamber to chamber after 2 (based on live cell images) and 48-h (based on stitched images of the whole device). ImageJ software (National Institutes of Health, Bethesda, MD, United States) was used to analyze the images.

#### 2.3.5 Immunocytochemical staining for cell-specific markers

Immunocytochemical staining for Vimentin (Abcam; ab92547; 1:300) in DECs, and Vimentin + Cytokeratin (CK)-18 (Abcam; ab668; 1:800) in AMCs and AECs, Histocompatibility Antigen (HLA)-G (Abcam; ab52455; 1:200) in CTCs, CK-7 (Abcam; ab9021; 1:600) in STBs and CTBs, and MUC18 (Abcam; ab233923; 1:200) in HUVECs were used as cell-specific markers. Antibodies were titrated to determine appropriate dilutions to ensure specific and uniform staining. After 24 h in culture, cells were fixed and permeabilized with 70% ethanol at 4C for 24 h. Before blocking, cells were washed 2x with 1× PBS and then blocked with 3% bovine serum albumin in 1× PBS for 1 h before incubation with primary antibodies overnight. Cells were washed three times in 1× PBS and then incubated with species-specific secondary antibodies (Abcam; Alexa Fluor 488-rabbit; ab150073) (Invitrogen; Alexa Fluor 594-mouse; A11005) (1:1000) for 1 h. Devices were washed with 1× PBS and then treated with NucBlue^®^ Fixed ReadyProbes Reagent (R37606; ThermoFisher Scientific, Waltham, MA) (2 drops per ml) to stain the nucleus and imaged as described below.

#### 2.3.6 Microscopy

Bright field microscopy or flouresence microscopy was performed (Nikon Eclipse TS100 microscope: ×10 magnification [bright field only] or Keyence All-in-one Fluorescence BZ-X810 microscope: ×2, ×10, and ×40 magnification) to determine cell morphology, expression of cell-specific markers, and 3000kd Dextran bead propagation.

#### 2.3.7 Drug propagation and metabolism across the FMi-PLA-OOC

Pravastatin sodium was used as a prototype drug. A physiological dose (200 ng/mL) of pravastatin obtained from the literature was used. The media from each reservoir, as well as the medium from the DEC chamber was removed, and pravastatin (200 ng/mL) was added to the decidual chamber and at different time points (0, 1 h, 2 h, 4 h, 8 h, and 24 h) supernatant from the different cellular chambers was collected and subjected to bioanalytical analysis.

### 2.4 Biological sample preparation and mass spectroscopy analysis

Frozen biological (collected supernatants) were thawed at room temperature and treated as follows: 100 μL of Internal standard solution (Rosuvastatin 400 ng/mL), 100 μL of the working solution for the calibration curve and QC samples, and 200 μL of Ice-cold methanol were added to 100-μL plasma samples. The mixture was vortexed for 5 min and centrifuged at 12,000 rpm for 10 min. The supernatant was transferred into a clean Eppendorf tube and evaporated to dryness under the stream of nitrogen. The residue was dissolved in 200 μL of the acetonitrile–water (50:50, v/v), vortexed, and centrifuged at 12,000 rpm for 15 min. The supernatant was passed through a 0.2-μm membrane filter, and 20 μL of the filtrate was injected for the LC-MS/MS analysis.

### 2.5 Mass spectrometry protocol - Targeted

Targeted liquid chromatography-tandem mass spectrometry triple quadrupole (LC-QQQ) analysis was performed on a TSQ Altis mass spectrometer (Thermo Scientific, Waltham, MA) coupled to a binary pump UHPLC (Vanquish, Thermo Scientific). Scan parameters for target ions were pravastatin—polarity negative, precursor m/z 423, products m/z 101, 303, and 321; The injection volume was 10 µL. Chromatographic separation was achieved on a Hypersil Gold 5 μm, 50 mm × 2.1 mm C18 column (Thermo Scientific) maintained at 30 °C using a solvent gradient method. Solvent A was 0.1% formic acid in water. Solvent B was 0.1% formic acid in acetonitrile. The gradient method used was 0–1 min (20% B to 60% B), 1–2 min (60% B to 95% B), 2–4 min (95% B), 4–4.1 min (95% B to 20% B), 4.1–5 min (20% B). The flow rate was 0.5 mL min-1. Sample acquisition and data analysis were performed Trace Finder 4.1 (Thermo Scientific).

#### 2.5.1 Mass spectrometry protocol—Untargeted

Untargeted liquid chromatography high-resolution accurate mass spectrometry (LC-HRAM) analysis was performed on a Q Exactive Plus mass spectrometer (Thermo Scientific, Waltham, MA) coupled to a binary pump UHPLC (UltiMate3000, Thermo Scientific). Full MS spectra were obtained at 70,000 resolution (200 m/z) with a scan range of 50–750 m/z. Full MS followed by ddMS2 scans were obtained at 35,000 (MS1) and 17,500 resolutions (MS2) with a 1.5 m/z isolation window and a stepped NCE (Vlčková et al., 2012; Nanovskaya et al., 2013; Richardson et al., 2019). Samples were maintained at 4°C before injection. The injection volume was 10 µL. Chromatographic separation was achieved on a Hypersil Gold 5 μm, 50 mm × 2.1 mm C18 column (Thermo Scientific) maintained at 30 °C using a solvent gradient method. Solvent A was 0.1% formic acid in water. Solvent B was 0.1% formic acid in acetonitrile. The gradient method used was 0–1 min (20% B to 60% B), 1–2 min (60% B to 95% B), 2–4 min (95% B), 4–4.1 min (95% B to 20% B), 4.1–5 min (20% B). The flow rate was 0.5 mL min-1. Sample acquisition was performed by Xcalibur (Thermo Scientific). Data analysis was performed with Compound Discoverer 3.1 (Thermo Scientific).

### 2.6 *In silico* drug modeling

#### 2.6.1 Software

All the modeling and simulations were conducted in GastroPlus™ (version 9.5, Simulations Plus, Inc., Lancaster, CA, United States), an *In silico* prediction tool for a comprehensive ADME description.

#### 2.6.2 Physicochemical properties

The chemical structure, physicochemical, biopharmaceutical, and pharmacokinetic parameters of pravastatin were obtained from the literature (García et al., 2003; Al-Badr and Mostafa, 2014; CID, 2021; Climent et al., 2021) and incorporated into the software. Based on the chemical structure, ADMET predictor™ module, we also determined the physio-chemical parameters that could be compared and refined as the observed data.

#### 2.6.3 Clinical pharmacokinetic data from literature

Clinical pharmacokinetic data of pravastatin in healthy subjects were taken from the work performed by Pan et al. (Pan et al., 1990) and Singhvi et al. (Singhvi et al., 1990). These reports described the mean plasma concentration-time curve of pravastatin after oral administration of 19.2 mg and 20 mg in humans. To obtain the values of the mean plasma concentration of pravastatin, the graphs were scanned with GetData Graph Digitizer (Version 2.26). Physiological values for intestinal volumes, lengths, and pH in humans were built into the software. The stomach transit time was changed to 0.1 h from 0.25 h as pravastatin was administered orally. The observed clinical data could be used to develop and validate the PBPK model of pravastatin. The methodology for developing the pregnancy PBPK model is depicted in Figure 1.

#### 2.6.4 Development and validation of the pravastatin PBPK model

The development and validation of the physiologically based pharmacokinetic (PBPK) model were divided into three significant steps: 1) Establishment of a nonpregnant PBPK model from observed data, 2) Validating the developed model with observed data, and 3) Applying the validated model to predict the pregnant PBPK model.

To establish a nonpregnant PBPK model, the pravastatin pharmacokinetics data were simulated using the Population Estimated for Age-Related Physiology (PEAR) module with a size of 20 virtual female subjects per population, randomly selected by the software with an average age between 20 and 40 years. The predicted mean values of the PK parameters C_max_ (highest plasma concentration), AUC (area under the curve), time at which the drug presents the highest concentration (T_max_), volume of distribution (V_d_), systemic clearance (CL), and half-life (T_1/2_) were verified with observed data (Costantine et al., 2013; Nanovskaya et al., 2013; Zarek et al., 2013; Al-Badr and Mostafa, 2014; van de Pas et al., 2014). The developed model was refined by optimizing parameters against clinical data from nonpregnant subjects and considered validated when there is no deviation more significant than 5%. Thus, the validated nonpregnant pravastatin PBPK model was developed.

The nonpregnant PBPK model represents the organs related to drug PK parameters like ADME. These organs are the heart, lung, brain, gut, spleen, gut, liver, kidney, adipose tissue, muscle, skin, and reproductive organs. These tissues are linked by arterial and venous blood; each compartment has its blood-flow rate, volume, and tissue-partition coefficient (Kp). Default values of Kp implemented in GastroPlus™ were used, which were calculated using the tissue composition equations according to the relationship between physiological data and compound-specific determinants of distribution-like lipophilicity (log P) (Al-Badr and Mostafa, 2014), ionization (pKa), and plasma protein binding (fu). PS is water soluble and majorly metabolized by glucuronidase in the stomach rather than cytochrome P450 in the liver. (Zhang et al., 2016). Although there is a slight effect of CYP3A4, the intrinsic clearance value was obtained from the literature and refined in the developed model. (Mao et al., 2018). The refined clearance values incorporated in the software were 30.00 ng/mL/min and 36.7 ng/mL/min for hepatic and renal excretion, respectively.

### 2.7 Statistical analyses

All data were analyzed using Prism 7 software (GraphPad Software, La Jolla, CA, United States). The Shapiro-Wilk test was conducted to check for the normality of the data. Student’s t-test was used to compare results with two means. Ordinary one-way analysis of variance followed by Tukey’s multiple comparison tests was used to compare normally distributed data with at least three means. The Kruskal–Wallis test with Dunn’s multiple comparison tests was used for data that were not normally distributed. Asterisks denote *p* values as follows: *p < 0.05; **p < 0.01; ***p < 0.001, ***p < 0.0001.

## 3 Results

### 3.1 Drug propagation and metabolism across both FMis using *in vitro* platforms

#### 3.1.1 Establishing a multi-organ fetal membrane-placenta feto-maternal interface on-chip model (FMi-PLA-OOC)

Two FMis, the fetal membrane, and placenta (Figure 1A) play critical roles in nutrient and oxygen transport, paracrine and endocrine signaling between the mother and the baby, performs barrier functions for exogenous substances, and maintain gestational tissue homeostasis (Aye et al., 2007; Behnia et al., 2015). The FMi tissues are often studied as explants (placenta or fetal membrane) or in a 2D culture system with dispersed cells from each tissue. These approaches reduce the ability to determine intercellular interactions and interface communications. In order to recreate both FMis in the FMi-PLA-OOC, the individual cell types were loaded into the device (i.e., design and fabrication methods 2.3.1) (Figures 1B, C), and cell morphology and cell-specific marker expression were evaluated (Figure 1D). AECs expressed an epithelial morphology and co-expressed vimentin (vim) and cytokeratin-18 (CK-18) within the FMi-PLA-OOC (Figure 1D). An elongated morphology and dominance of vim were seen in AMCs confirming their mesenchymal nature on-chip (Figure 1D). CTCs remained cuboidal and expressed the immune regulatory marker Human leukocyte antigen-G (HLA-G) within the cell chamber (Figure 1D). While DECs expressed an elongated morphology and dominance of vim typical of the decidua (Figure 1D). STB cultured on-chip maintained their epithelial morphology, contained multiple nuclei and microvilli and had decreased cytokeratin-7 (CK-7) expression, confirming syncitialization had occurred (Figure 1D). CTBs maintained their epithelioid morphology and CK-7 expression (Figure 1D). HUVEC cultured within the FMi-PLA-OOC contained an endothelial morphology and expressed cell adhesion molecule MUC18 (Figure 1D). These results validate that the cellular components of the model retain their key characteristics as seen *in utero*. To evaluate the utility of the multi-organ FMi-PLA-OOC to conduct preclinical drug testing, pravastatin kinetics and metabolism across the fetal membrane and placenta layers were assessed.

#### 3.1.2 Propagation of pravastatin across the fetal membrane and placenta layers

**FIGURE 2:**
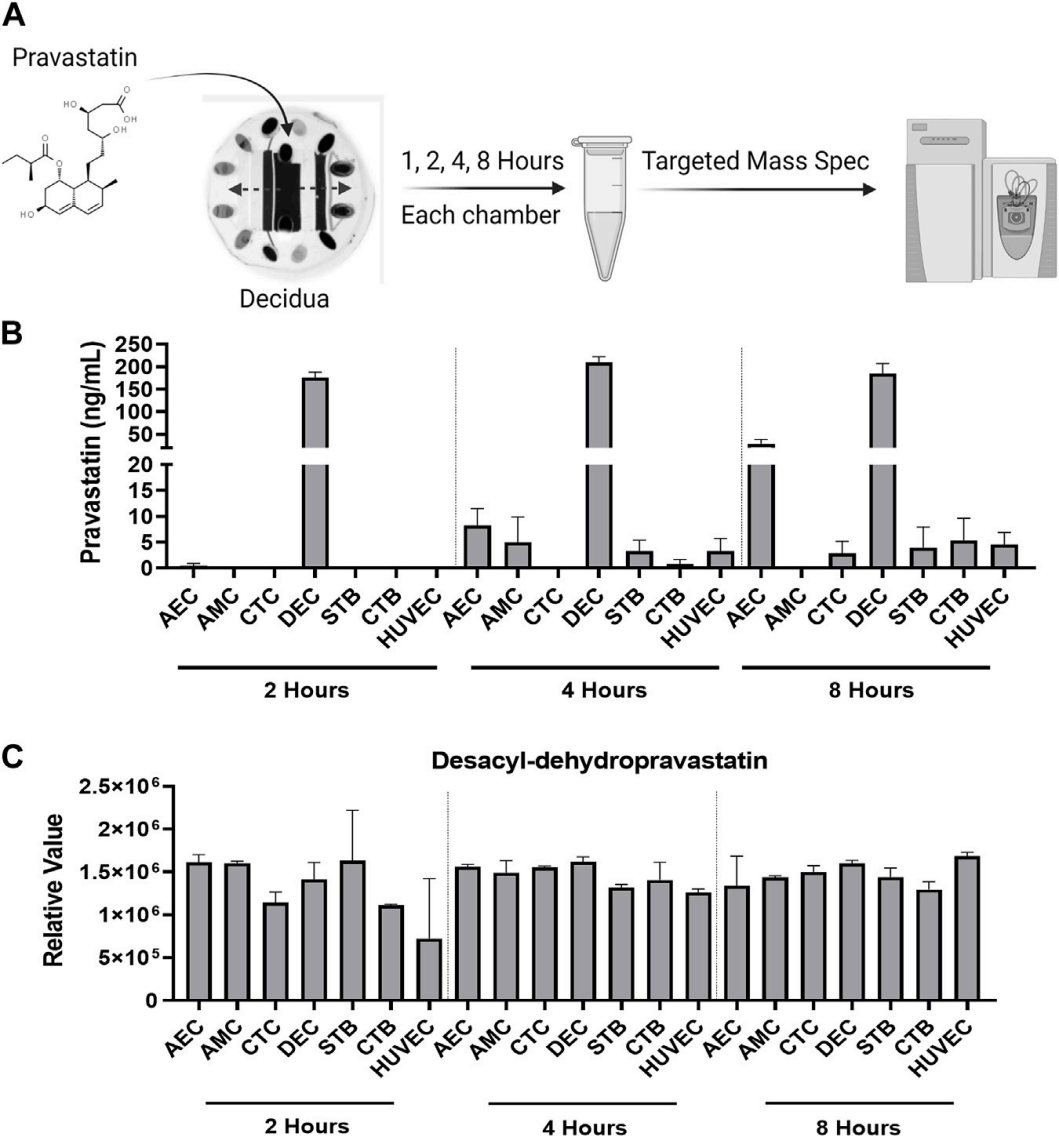
Pravastatin pharmacokinetics across the Fetal Membrane Placenta layers **(A)** Schematic workflow of pravastatin treatment within the DEC chamber of the FMi-PLA-OOC. **(B)** Targeted mass spectrometry showed that Pravastatin can cross the FMi-PLA-OOC within 4 h and these values increase over time. Values are expressed as mean intensities ±SEM (N = 3–4). **(C)** Untargeted mass spectrometry identified pravastatin metabolite, Desacyl-dehydropravastatin, that can cross the FMi-PLA-OOC within 2 h. Values are expressed as mean intensities ±SEM (N = 3).

Within the FMi-PLA-OOC, we evaluated fluid diffusion characteristics between cell chambers to establish baseline values. The ability to maintain fluidic separation between the seven chambers of the FMi-PLA-OOC was tested using Texas Red 3000kd beads. Beads were introduced into the DEC chamber and reached the adjacent CTC and STB cells within 2 h; however, these beads did not transverse through the other cell layers until after 48 h (Supplementary Figure S1). Thus, due to these results and our previous single organ (i.e., FMi-OOC and PLA-OOC) pravastatin propagation kinetics studies (Richardson et al., 2022), experimental time points of 2, 4, and 8 h were set to determine pravastatin kinetics across the fetal membrane and placenta FMis. To determine statin pharmacokinetics over time, a therapeutic concentration (200 ng/mL) of pravastatin that does affect cell viability (Richardson et al., 2022) was added to the DEC. Media were collected from each chamber and analyzed by targeted mass spectrometry, as depicted in Figure 2A. Pravastatin reached the AEC (8.2 ± 3.3 ng/mL) and HUVEC cells (3.3 ± 2.4 ng/mL), the farthest from the DEC, within 4 h (Figure 2B). The concentrations increased by 8 h, specifically in the AECs (28.8 ± 9.3 ng/mL) (Figure 2B). Additionally, we determined that each cell type within the placenta and fetal membrane can metabolize the parent compound in a time and cell-type-specific manner.

#### 3.1.3 Pravastatin metabolism within the FMi-PLA-OOC

Pravastatin exhibits unique metabolic characteristics due to its composition and utilization of cell-specific enzymes. It can quickly be broken down into various inactive metabolites, including 3′α-Iso pravastatin, 6′-Epipravastatin, 3′α, 5′β-Dihydro-pravastatin, Desacyl-dehydropravastatin, or 3′-Hydroxy-pravastatin and excreted from the human body (Zhang et al., 2016). Pravastatin primarily undergoes glucuronidation during metabolism instead of relying on cytochrome P450 (CYP) enzymes (Vlčková et al., 2012; Zarek et al., 2013; Zhang et al., 2016). To investigate the metabolism of pravastatin within different cellular layers of the fetal membrane and placenta, the media from the FMi-PLA-OOC chambers were analyzed using untargeted mass spectrometry. After 4, 8, or 24 h, it was observed that all cell layers metabolized pravastatin into low levels of Desacyl-dehydropravastain within 2 h. In certain cell layers, the levels of Desacyl-dehydropravastatin increased over time (i.e., HUVECs) (Figure 2C), while in other cell layers, it remained constant. These findings indicate that the fetal membrane and placental cells have the capability to metabolize pravastatin and convert it into its inactive form.

### 3.2 Simulation of drug pharmacokinetics utilizing GastroPlus^®^ simulation software

**FIGURE 3:**
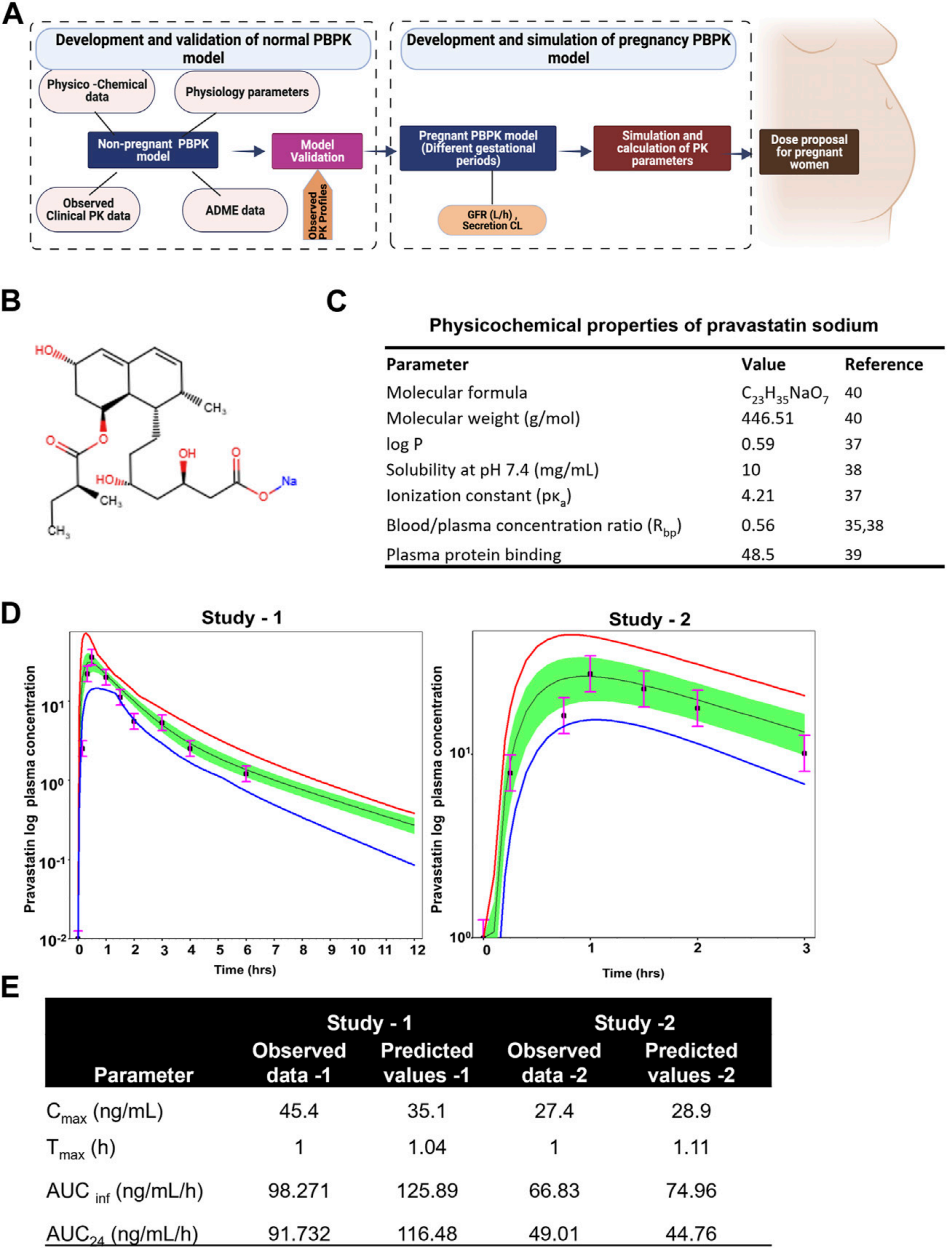
Developing and validating the pravastatin pharmacokinetics in nonpregnant population. **(A)** Structure of developing pregnancy PBPK model **(B)** Chemical structure of pravastatin sodium. Chemical structure obtained from PUBCHEM **(C)** Physicochemical properties of pravastatin sodium **(D)** Predicted and observed plasma semi-log concentration-time profile of pravastatin after administration of single oral dose at 20 mg and 19.2 to normal healthy volunteers. The black circle symbols represent the mean of observed data and the solid black line represent the predict mean of pravastatin profiles. Green shaded areas represent the 90% confidence interval for the simulated data. The blue and red lines are 95% probability lower and upper limits of predicted values. **(E)** Predicted and observed pharmacokinetics parameters in normal healthy volunteers after single dose of pravastatin at 20 mg.

The illustration of the developing pregnancy-related PBPK model was depicted in the Figure 3A.

#### 3.2.1 Physicochemical properties and observed clinical pharmacokinetics

Pravastatin sodium is an organic sodium salt and is used clinically as a cholesterol-lowering agent. It is a high polarity compound and its solubility in water at pH7.2 is 10 mg/mL. The ionization constant of pravastatin is reported as 4.2 (pKa value) and logp value as 0.59 (49). Pravastatin pharmacokinetic parameters (C_max_ and T_max_) in a Caucasian population at an oral dose of 19.2 mg were reported as 27.4 ± 10.7 ng/mL as 0.88 ± 0.16h, respectively. The oral bioavailability of pravastatin was reported to be nearly 18%, with substantial biliary excretion. The average renal clearance of pravastatin is greater than 400 mL/min, which indicates that tubular secretion is the predominant mechanism in renal excretion. Pan et al., assessed the effect of age on the pharmacokinetics of pravastatin in men and women (Pan et al., 1990; Pan et al., 1993). At an oral dose of 20 mg pravastatin, C_max_ was 45.4 ± 8.4 and 42.1 ± 5.3 ng/mL at T_max_ 1.0 ± 0.1 and 1.2 ± 0.10 h for younger and elderly populations, respectively. The protein binding percentage was 54.5%, with 7.5% ± 1.2% urinary excretion. No difference in the disposition of pravastatin between men and women and only small differences between young and elderly subjects were reported (Pan et al., 1993). The chemical structure and summary of physicochemical parameters used in this study are shown in Figures 3B, C.

#### 3.2.2 Development and validation of the PBPK model for pravastatin

By incorporating the physico-chemical parameters of pravastatin, we successfully simulated the reported nonpregnant pharmacokinetic data using the ADMET predictor module within the simulation software. The developed nonpregnant PBPK model was validated against two different pravastatin pharmacokinetic studies reported by Sanghvi et al. and Pan et al. The developed and validated PBPK model for pravastatin in normal healthy adults is shown in Figure 3D. The simulated pharmacokinetic data from the software agreed with the observed values by comparing the values like (C_max_, T_max_, AUC_0-t_, and AUC_0-∞_). The fold error of the simulated data was within the 95% confidence interval of prediction, and the developed PBPK method can be used to simulate pregnancy PBPK parameters of PS. The observed and simulated pravastatin values showed in Figure 3E.

#### 3.2.3 Development of pravastatin pregnancy-related PBPK model

**FIGURE 4:**
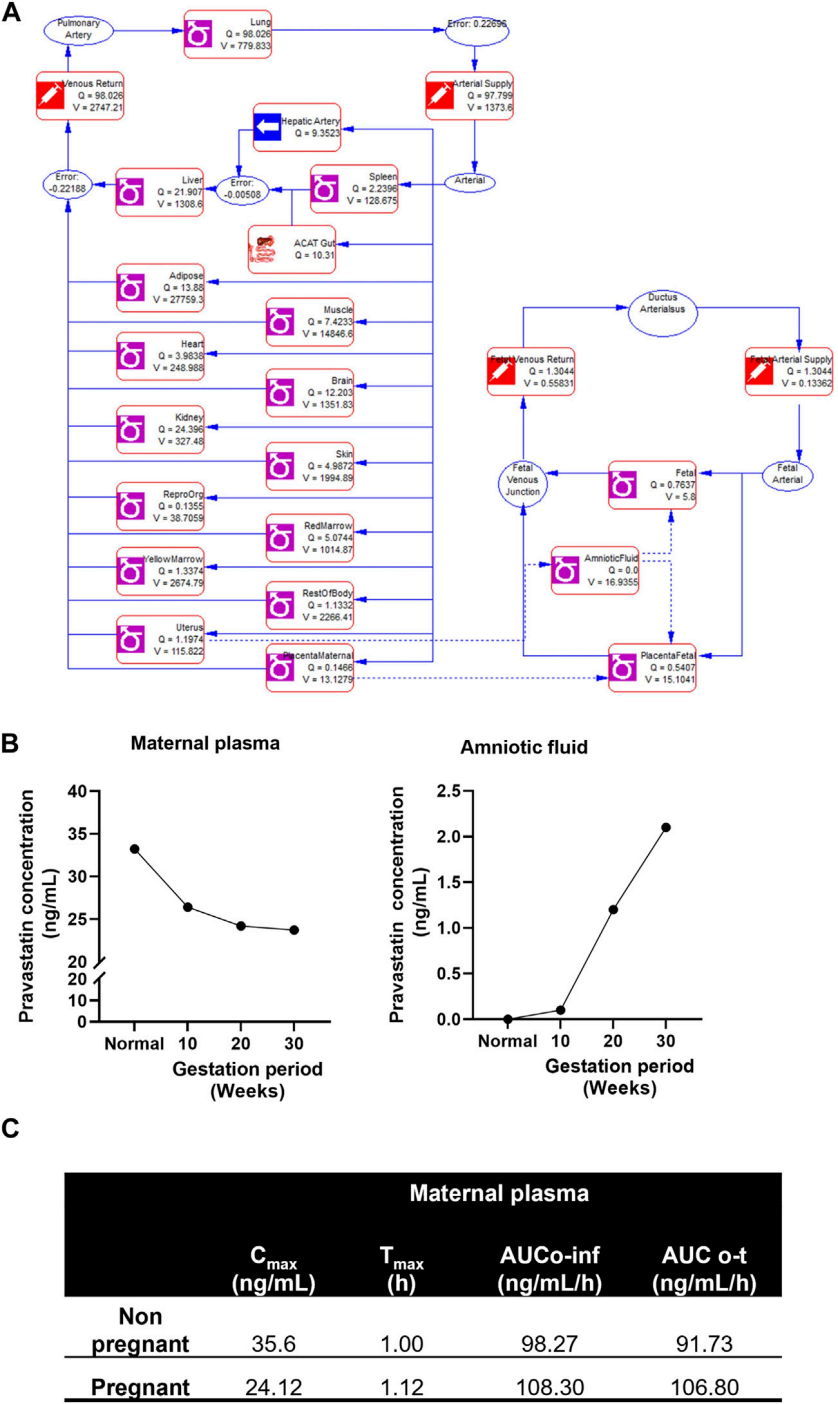
Simulated pregnant pharmacokinetics of pravastatin. **(A)** Structure of pregnancy related PBPK model. Structure of Pregnancy PBPK was obtained from Gastroplus software. **(B)** Predicted pharmacokinetics of pravastatin concentrations in maternal plasma and amniotic fluid during different gestation ages. **(C)** Simulated pharmacokinetics of pravastatin in maternal plasma and comparison to nonpregnant population.

The structure of the pregnancy-related PBPK model is depicted in Figure 4A. To develop a PBPK model for the pregnant population over the three trimesters, we extended the validated nonpregnant model to predict pregnancy-related pharmacokinetics by incorporating the fetal-maternal compartments, including maternal placenta, fetal placenta, amniotic fluid, and fetal venous system (as shown in Figure 4A). The software adjusted the intrinsic clearance rates of the fetal and maternal tissues based on the nonpregnant pharmacokinetic data. The concentration-time profile of maternal plasma and amniotic fluid showed a decrease in the plasma concentration of the pregnant population (24.12 ± 6.32 ng/mL) compared to the nonpregnant population (35.6 ± 6.32 ng/mL) and an increase in pravastatin concentration in the amniotic fluid as gestation progressed. (Figure 4B). and the simulated pharmacokinetic parameters of the pregnant population showed maximum time (T_max_) to reach maximum concentration was 1.12 + 0.30 h and total drug exposure across time (AUC_0-∞_) was 108.30 + 12.31 ng/mL/h (Figure 4C). The physiological changes during pregnancy were considered, and data were simulated. The simulated data using GastroPlus^®^ exhibited drug transport across the placenta, consistent with findings from placenta perfusion studies conducted by Costantine et al. (Costantine et al., 2016; Costantine et al., 2021). These findings suggest that developing models that mimic the three trimesters of gestation could be utilized to predict pregnancy pharmacokinetics, thus resembling the clinical scenario.

### 3.3 Comparison of results from the FMi-PLA-OOC and simulation model

**FIGURE 5:**
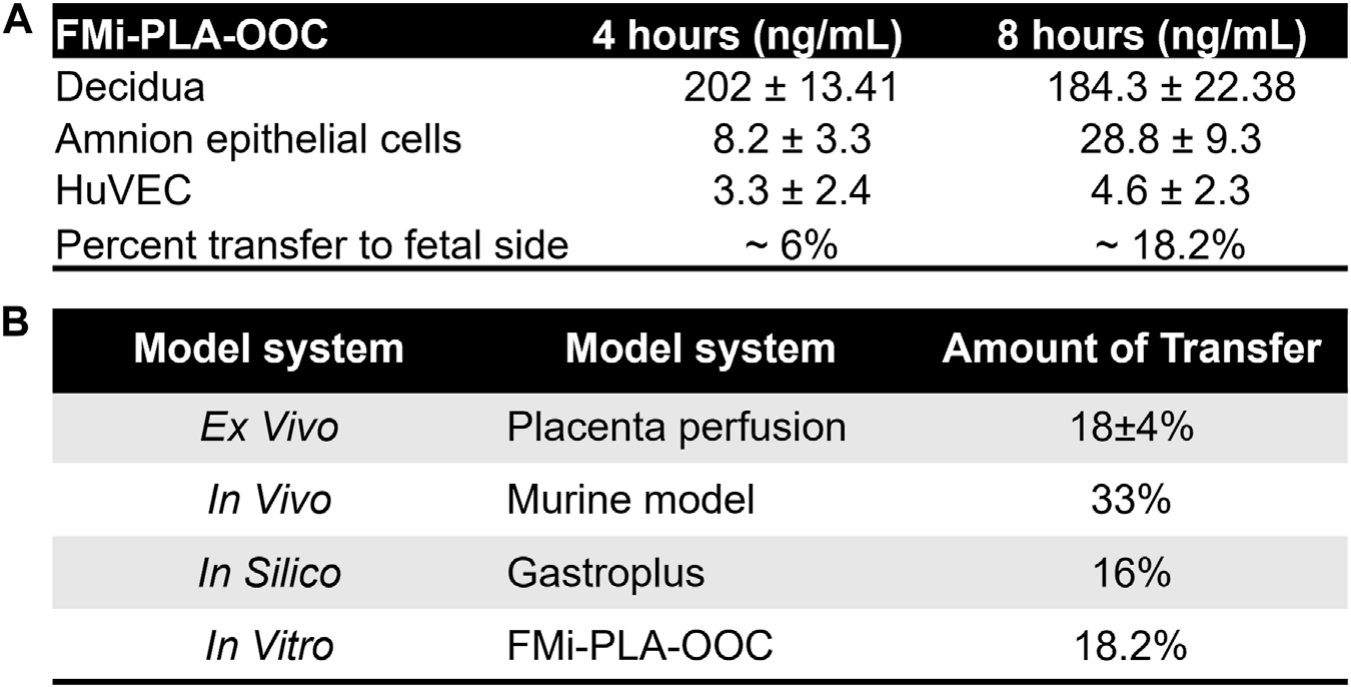
Validation of FMi-PLA-OOC and simulation results **(A)** Concentration of pravastatin that propagates across the FMi-PLA-OCC at 4 and 8 h in Amnion and endothelial cells. **(B)** Comparison of results from the FLA-PLA-OOC and simulation model to placental perfusion and *In vivo* pregnant mouse model.

To validate and establish the biological relevance of the drug transfer rates obtained from the FMi-PLA-OOC system, we compared the results with published placenta perfusion data (Costantine et al., 2013; Nanovskaya et al., 2013; Zarek et al., 2013; Costantine et al., 2016; Costantine et al., 2021)as well as data from *in silico* simulation models (Figure 3) and *in vivo* mouse models (Supplementary Figure S2). In the FMi-PLA-OOC, the percentage of drug transfer from the maternal decidual to the fetal amnion or HUVEC side was approximately 6% ± 1.8% and 18% ± 2.3% within 4 and 8 h, respectively (Figure 5A). Compared to placenta perfusion models, the drug transfer data obtained from the FMi-PLA-OOC (18.2%) and simulation models (16%) exhibited closer alignment to the clinical scenario. In contrast, the data from animal models did not closely resemble the human-based data, with a transfer rate of approximately 33% in mice models (Figure 5B). These findings highlight the potential utility of microfluidic and simulation platforms in predicting and determining the pharmacokinetics of drugs during pregnancy.

## 4 Discussion

Despite the rise in maternal morbidity and mortality, as well as the unique physiologic characteristics of people during pregnancy, pregnant and lactating people remain therapeutic orphans because they are excluded from the vast majority of clinical drug development and therapeutic trials. (Sheffield et al., 2014; ACoOa, 2015; Zimmerman et al., 2015; Illamola et al., 2017; McKiever et al., 2020). Pregnant women represent the most therapeutically vulnerable population as evident during disease outbreaks and pandemics like COVID-19 (Atyeo et al., 2022; Blakeway et al., 2022). Treating pregnant women affects the maternal and fetal systems and have implications for the next-generation’s health. However, there is a significant lack of knowledge regarding the pharmacokinetics of drugs, both prescribed and over the counter, taken during pregnancy (Buhimschi and Weiner, 2009; Cleary et al., 2014; Costantine, 2014). Unfortunately, conducting pharmacokinetic studies during pregnancy poses challenges for pharmaceutical industries due to ethical considerations, a relatively small market, and the complexity, time requirements and high costs associated with experimental models (McKiever et al., 2020; David et al., 2022). As a result, reliable models for studying drug behavior during pregnancy are limited. To address these limitations, we have assessed and validated the FMi-PLA-OOC for conducting preclinical drug testing. In our study, we focused on comparing pravastatin transfer rates within *in vitro* FMi-PLA-OOC, *in silico* simulation Gastroplus^®^ (generated from existing literature (Halstenson et al., 1992; Hatanaka, 2000; Kyrklund et al., 2004; Kivistö et al., 2005; Costantine et al., 2016; Li et al., 2017; Costantine et al., 2021)), *ex vivo* placental perfusion, and *in vivo* mouse models. As pravastatin is currently being considered as a clinical treatment for preeclampsia, providing information regarding its rate of transfer and metabolism across both FMis, along with simulation-PBPK parameters at different gestational ages, will be valuable for clinical researchers seeking to understand its behavior. Together, we propose that the combination of microfluidic technology and artificial intelligence-based simulation models could provide a reliable alternative to expensive *in vivo* experiments for studying drug pharmacokinetics during pregnancy. They also provide a more reliable and human-relevant approach than animal models and a better platform for preclinical drug testing than placenta perfusion (i.e., the need for healthy and disease platforms). Our data support the development of non-animal model preclinical drug testing platforms in accordance with the US FDA Modernization Act 2.0.

In previous studies, we have reported similar expression patterns of transporter proteins and cytochrome P450 enzymes in fetal membranes and placental tissues (Ganguly et al., 2021; Kammala et al., 2021; Kammala AK. et al., 2022; Kammala A. et al., 2022). Our previous fetal membrane and placenta specific OOC models have been to test the statin drug propagation and established the role of drug transporters and metabolic enzymes (Ganguly et al., 2021; Richardson et al., 2022). Pravastatin transportation involves various drug transporter proteins, such as OATPs, BCRP-1, P-gp, and MRP-1. However, we observed that higher amounts of pravastatin propagated to the fetal amnion side compared to the placental endothelial cells. This suggests that pravastatin transportation in cells across the device is primarily carried out by drug transporter proteins rather than passive diffusion (Komai et al., 1992a; Komai et al., 1992b; Afrouzian et al., 2018; Deng et al., 2021). These findings further support our previous results on statin transport across various fetal membrane cell layers using a different organ chip models (Ganguly et al., 2021). Additionally, compared to the placenta, fetal membranes showed higher pravastatin transportation, indicating the importance of fetal membranes in drug transportation studies. Therefore, devices like FMi-PLA-OOC, which incorporate both the placenta and fetal membranes, are essential for determining pregnancy-associated pharmacokinetics and studying drug transporters and their function.

Furthermore, in this study, we investigated the metabolism of pravastatin across both FMis. The FMi-PLA-OOC can be used to determine known and unknown metabolites of drugs produced by different cells in the placenta and fetal membranes. This provides an opportunity to discover active metabolites of drugs and aids in the development of novel drug delivery strategies for pregnancy-related disorders. This represents a breakthrough in pregnancy-related drug testing, as most drug metabolism studies are typically conducted in hepatocytes (Ziegler and Hummelsiep, 1993; Deng et al., 2021), neglecting the metabolism of drugs that bypass the first-pass hepatic system during pregnancy. Although, not shown in this study, but reported already, besides kinetics and metabolism of various compounds, FMi-PLA-OOCs can determine efficacy to revert a disease phenotype of the fetal membranes and the placenta into a healthy state, cytotoxicity, and rate of absorption by cells. To note, this study neither validate nor propose the use of pravastatin use during pregnancy, but we used this drug as a model to test our OOC model as multiple sets of data are available for this drug during pregnancy for our comparisons.

To comply with the FDA Modernization Act 2.0, which promotes the use of alternatives to animal testing for drug development and testing (FDA, 2022), we employed the Gastroplus^®^ simulation software to predict maternal drug pharmacokinetic parameters at different gestational ages. By integrating *in silico* simulation software like Gastroplus® with relevant physiological parameters and experimental data, we can obtain valuable insights into the pharmacokinetics of drugs during pregnancy. This integration of *in silico* and experimental data offers a practical and efficient means of studying drug behavior in pregnant individuals, holding substantial promise in advancing drug transport research during pregnancy. Such advancement contribute to the development of safer and more effective pharmacotherapy for this vulnerable population, aligning with the FDA’s commitment to promoting the adoption of alternative approaches in drug development and ensuring the safety and efficacy of medications for pregnant individuals.

Further, data from FMi-PLA-OOC (18% ± 2.3% of drug transfer) and simulation model (16%) mimic the placental perfusion studies (∼18% ± 6%) and the clinical pharmacokinetics data. Placental perfusion models have been used traditionally for preclinical drug testing. These models have been helpful as they and involve the exchange of blood and drug transportation across the placenta; however, several limitations of this model have hindered regulatory approvals of drugs (Elbæk Madsen et al., 2019) (**Table 1**) Some of these limitations include: 1) Ethical considerations: Even though there is no direct involvement of human subjects, obtaining placental tissue after delivery requires ethical considerations for placental perfusion studies. On the other hand, simulation models rely on computer simulations, and OOC technology is based on human cells, making them ethically advantageous for studying human physiology. 2) Cost and time efficiency: Placental perfusion studies were time-consuming and required specialized equipment and expertise, similar to animal studies. 3) Physiological relevance: placental perfusion studies are often conducted using *ex vivo* placental tissue obtained after delivery. While these studies provide insights into the placental transport of a compound, the results may not fully represent the *in vivo* conditions during pregnancy (Berveiller et al., 2017). Factors such as labor, delivery, and maternal health status at the time of delivery can influence placental function. 4) Limitation to conduct to large-scale studies: the process of setting up the perfusion system, establishing circulation, and conducting measurements can be time-consuming. This limits the number of samples that can be processed within a given time frame and conducting large-scale studies are difficult. 5) Physiological challenges: Placental perfusion models are restricted to term placenta that has undergone massive biological, mechanical, immunological and endocrinological changes associated with pregnancy termination at term and does not represent a placenta (first, second or third trimester) when a drug is expected to be administered during pregnancy. 6) Cell biological challenges: Term placenta shows multitudes of phenotypes of cell deaths, including necrosis, senescence, and apoptosis (Giaginis et al., 2011; Myllynen and Vähäkangas, 2013; Berveiller et al., 2017). These cell death factors can confound drug transport function. A disease state of the placenta (e.g., placentitis, chorioamnionitis, oxidative stress, immune cell infiltration, microbial presence state) cannot be replicated in perfusion models. Baseline pharmacokinetics data generated from normal term placenta may not reflect placental kinetics functions for the same drug during a disease state. 7) Confounding variables: Subject to subject variability of the placental cellularity and biology, pregnancy-associated changes, and clinical and demographic variations can impact placental perfusion data. 8) Restriction to data acquisition: Perfusion models are still ideal for measuring the baseline transport kinetics of a compound, but that is also the limit of its use. The efficacy of a drug, the impact of cytotoxicity over a period of exposure, metabolism at different cell levels, mechanism of action, and molecular and functional biology of the placenta in response to a drug are difficult to determine using this model system. 9) Difficulty in interpreting results: the interpretation of placental perfusion study results can be complex. There may be variations in the placentas, approaches of perfusion techniques, equipment, and protocols used across different research studies, making it challenging to compare and combine data from different sources (Giaginis et al., 2011). Additionally, the relationship between perfusion measurements and specific clinical outcomes may not be fully understood, leading to uncertainties in clinical applicability (Myllynen and Vähäkangas, 2013).

**TABLE 1:** Comparison of *in vitro, in silico,* and *in vivo* model systems.

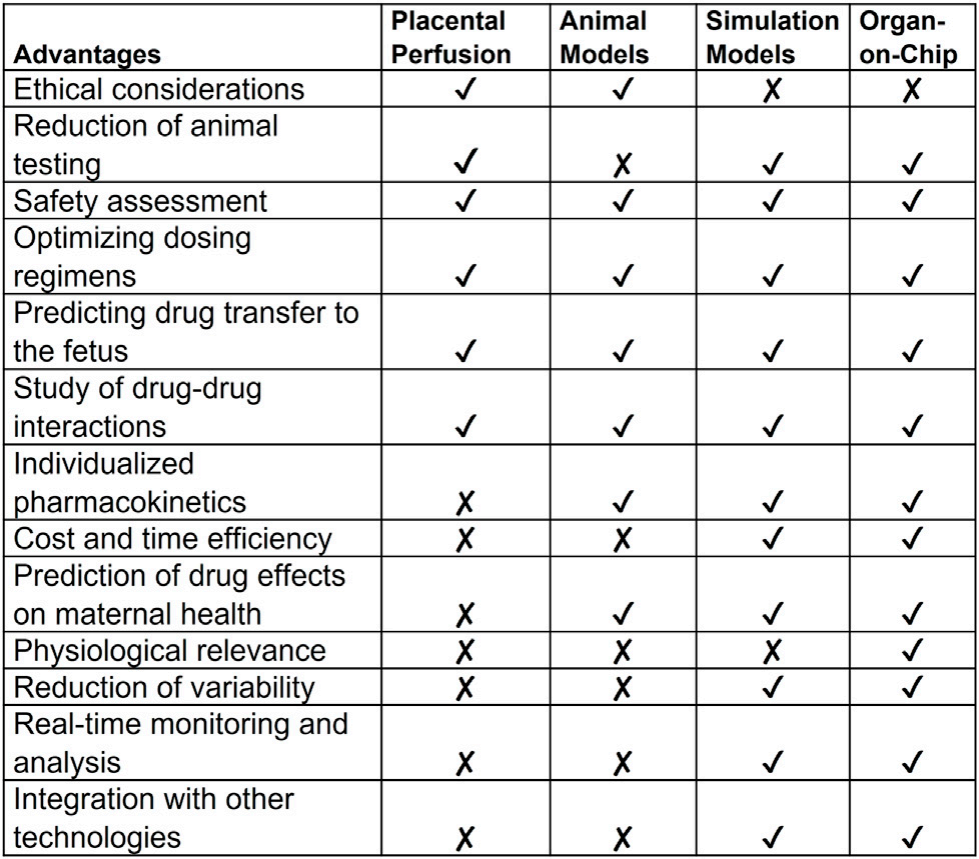

Conversely, microfluidic OOC technology has several advantages (**Table 1**) by replicating the structure and function of human organs in a microscale format that allows overcoming many of the limitations (Arumugasaamy et al., 2020). OOC models mimic accurately the physiological conditions of specific organs and different disease states associated with pregnancy conditions. The normal and disease states can be established prior to testing a compound. OOCs can determine efficacy, metabolism, absorption, and toxicity. The scalability of OOCs allows high throughput data generation in a very short time for a thorough analysis of functional and pharmaco-biological changes of the organs. Mechanistic modeling, functional studies, testing specific enzyme or receptor contribution can be easily modeled. Dynamic flow setup can facilitate blood/fluid flow and this can be easily done using syringes and pumps (not tested in our current models). OOC allows high-throughput screening that enables simultaneous testing of multiple drugs or compounds. This accelerates the drug discovery process and reduces costs associated with traditional screening methods. OOCs can avoid interindividual variations by using well characterized and functionally proven cell lines or induced pluripotent stem cells. Compared to placental perfusion models, OOC technology has the potential to contribute the personalized medicine and can be tailored to incorporate patient-specific cells or disease models, allowing for the testing of individualized drug responses and the development of personalized treatment strategies. By incorporating other technologies like biosensors and imaging techniques, OOC models can be used to study the real-time monitoring of cellular responses and physiological parameters at the FMis. This provides researchers with valuable data on tissue function, drug effects, and disease progression in different pregnancy-related disorders. Overall, OOC technology and simulation software hold great promise for advancing biomedical research, drug development, and personalized medicine, providing more physiologically relevant models for studying human pregnancy.

OOCs are potential alternatives to animal testing, providing a platform for studying human physiology and disease mechanisms (Richardson L. et al., 2020). They can simulate human organ responses to drugs, toxins, and diseases, thereby reducing the reliance on animal models and enhancing ethical considerations. In combination with simulation software like Gastroplus^®^, OOCs models can predict drug pharmacokinetics and could be able to determine drug efficacy and safety. It helps to reduce the risk of adverse effects in human clinical trials. Limitations of our study include the lack of direct assessment of the risk or consequences of drugs on the fetal or maternal side, which falls beyond the scope of this manuscript. While we have focused on drug transport across the two feto-maternal barriers, further investigations are needed to fully understand the short- and long-term consequences of these drugs. Additionally, our study primarily utilized pravastatin as a model drug, and while it provided valuable data for comparisons, other drugs may exhibit different transport behaviors in the FMi-PLA-OOC platform. Furthermore, our study did not address the potential transgenerational impacts of drug exposure during pregnancy, which may necessitate the use of animal models for further investigations. Despite these limitations, our findings contribute to the advancement of non-animal model preclinical drug testing platforms, aligning with the FDA’s initiative to promote alternative approaches in drug development for pregnant individuals. The combination of microfluidic OOC technology and simulation software offers a more human-relevant and cost-effective approach to studying drug behavior during pregnancy, providing valuable insights for the development of safer and more effective pharmacotherapy for this vulnerable population.

## 5 Conclusion

In conclusion, use of microfluidic devices like the FMi-PLA-OOC offers a practical approach for studying drug transportation and metabolism across FMis during pregnancy within an *in vitro* setting. Additionally, the integration of mathematical-based *in silico* simulation software enables the development of PBPK models, incorporating prior knowledge from *in vitro* and *in vivo* data. These models can successfully predict drug pharmacokinetics at different gestational ages and in multiple populations. This study highlights the role of FMi-PLA-OOC and pregnancy-related pharmacokinetics in drug development, particularly when obtaining experimental data is challenging or limited. The combination of microfluidic devices and *in silico* modeling provides a promising alternative to traditional approaches, offering a cost-effective and ethically sound platform for assessing drug behavior during pregnancy. By understanding the drug transportation and metabolism across FMis, this research contributes to improving drug safety and efficacy for pregnant women and their developing fetuses.

## Data Availability

The original contributions presented in the study are included in the article/Supplementary Material, further inquiries can be directed to the corresponding authors.
