# Supplementary figures and images for: Microfluidic technology and simulation models in studying pharmacokinetics during pregnancy
Source: Front Pharmacol. 2023 Aug 17;14:1241815. doi: 10.3389/fphar.2023.1241815 (PMC10469630; doi:10.3389/fphar.2023.1241815)

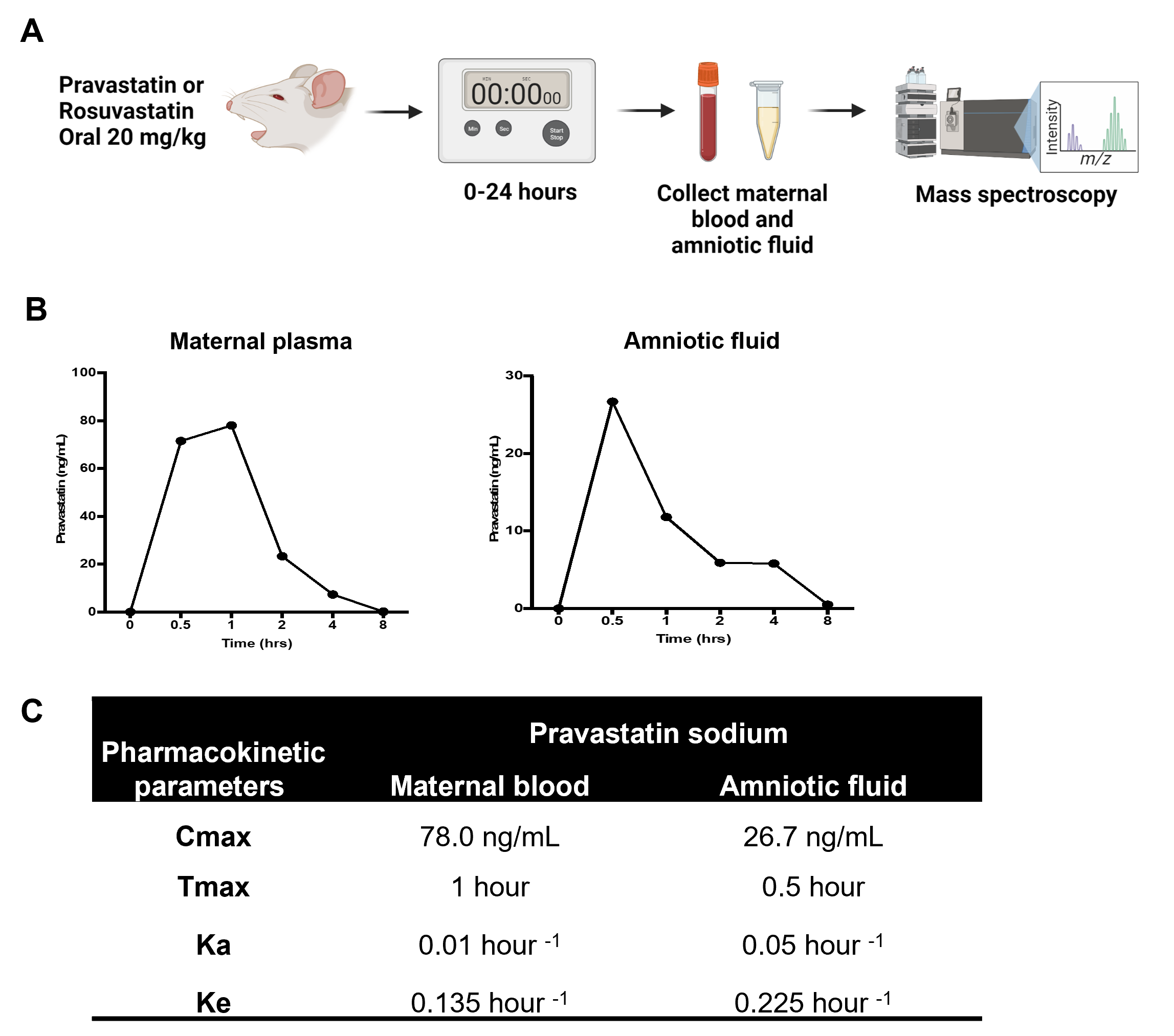

Supplement: Supplementary file 1 [file Image2.TIF]

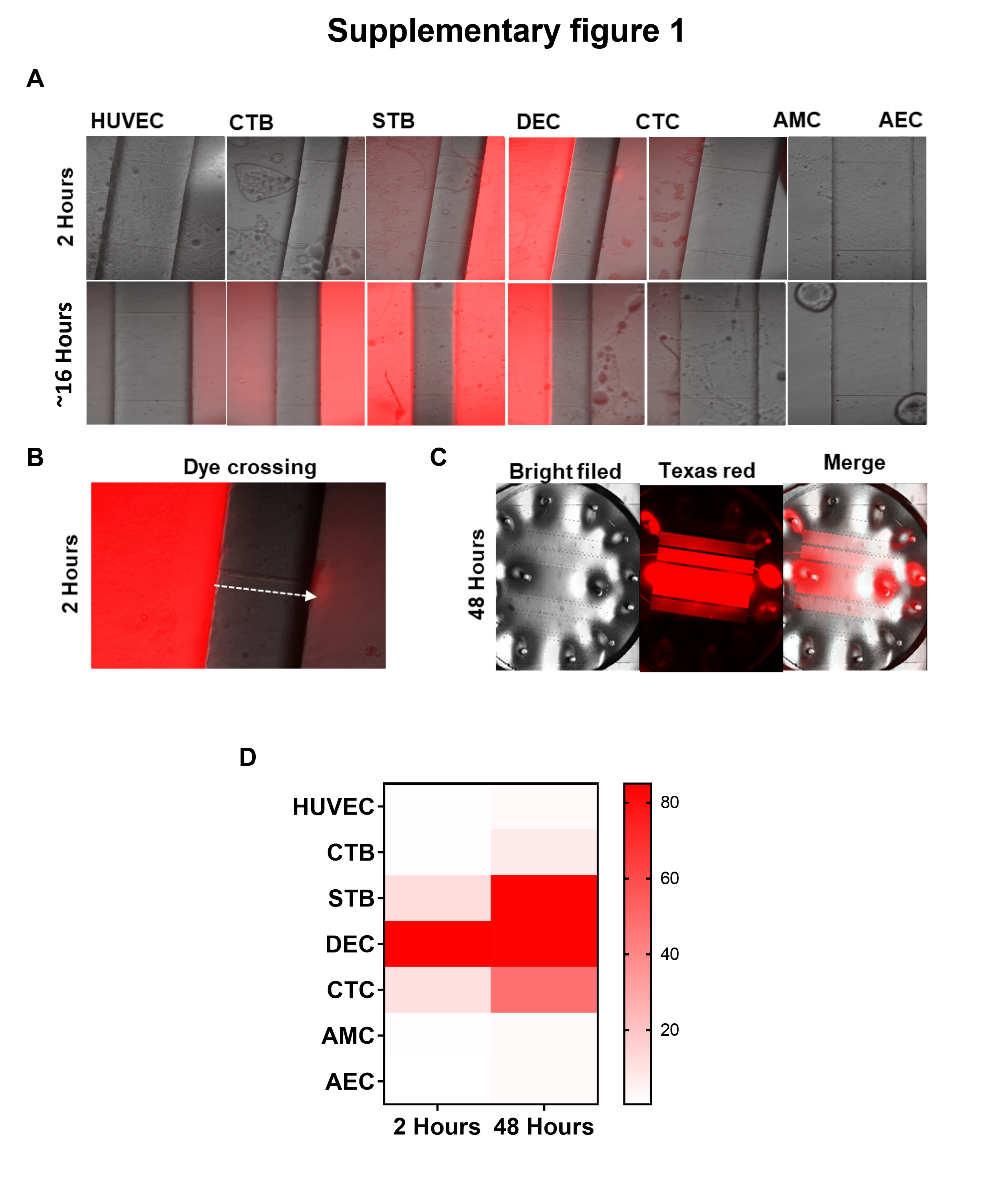

Supplement: Supplementary file 2 [file Image1.TIF]
